# Chitosan nanoparticles modified TLC-densitometry for determination of imidacloprid and deltamethrin residues in plants: greenness assessment

**DOI:** 10.1186/s13065-023-00941-2

**Published:** 2023-04-04

**Authors:** Ghadeer A. Elbaz, Hala E. Zaazaa, Hany H. Monir, Lobna M. Abd El Halim

**Affiliations:** 1 Pharmaceutical Chemistry Department, Egyptian Drug Authority, Giza, Egypt; 2 Analytical Chemistry Department, Faculty of Pharmacy, Cairo University, Kasr El Aini Street, Cairo, 11562 Egypt

**Keywords:** Pesticides residues, Imidacloprid, Deltamethrin, Thyme and guava leaves

## Abstract

Two thin layer chromatography (TLC) methods have been developed for the determination of pesticides residues of imidacloprid (IMD) and deltamethrin (DLM) in thyme and guava leaves. In the two methods, the used stationary phase was silica gel 60 F_254_ plates impregnated in chitosan nanoparticles (ChTNPs) 0.5% to improve separation using a green developing system consists of isopropyl alcohol for IMD and *n*-hexane-toluene-ethylacetate for DLM. The two pesticides were determined quantitatively, after TLC separation, at wavelengths 270.0 nm for IMD and 230.0 nm for DLM. Validation of both approaches was carried out in agreement with the guidelines of International Conference on Harmonization (ICH) and found to be selective, reliable and reproducible. Limits of detection of IMD and DLM were 0.002 and 0.00116 μg/spot, respectively. The newly developed TLC methods were used to monitor the pre-harvest interval estimation. Analytical eco-scaling depending on penalty points for IMD was calculated and showed that this method was eco-friendlier than the reported one.

**Graphical Abstract:**

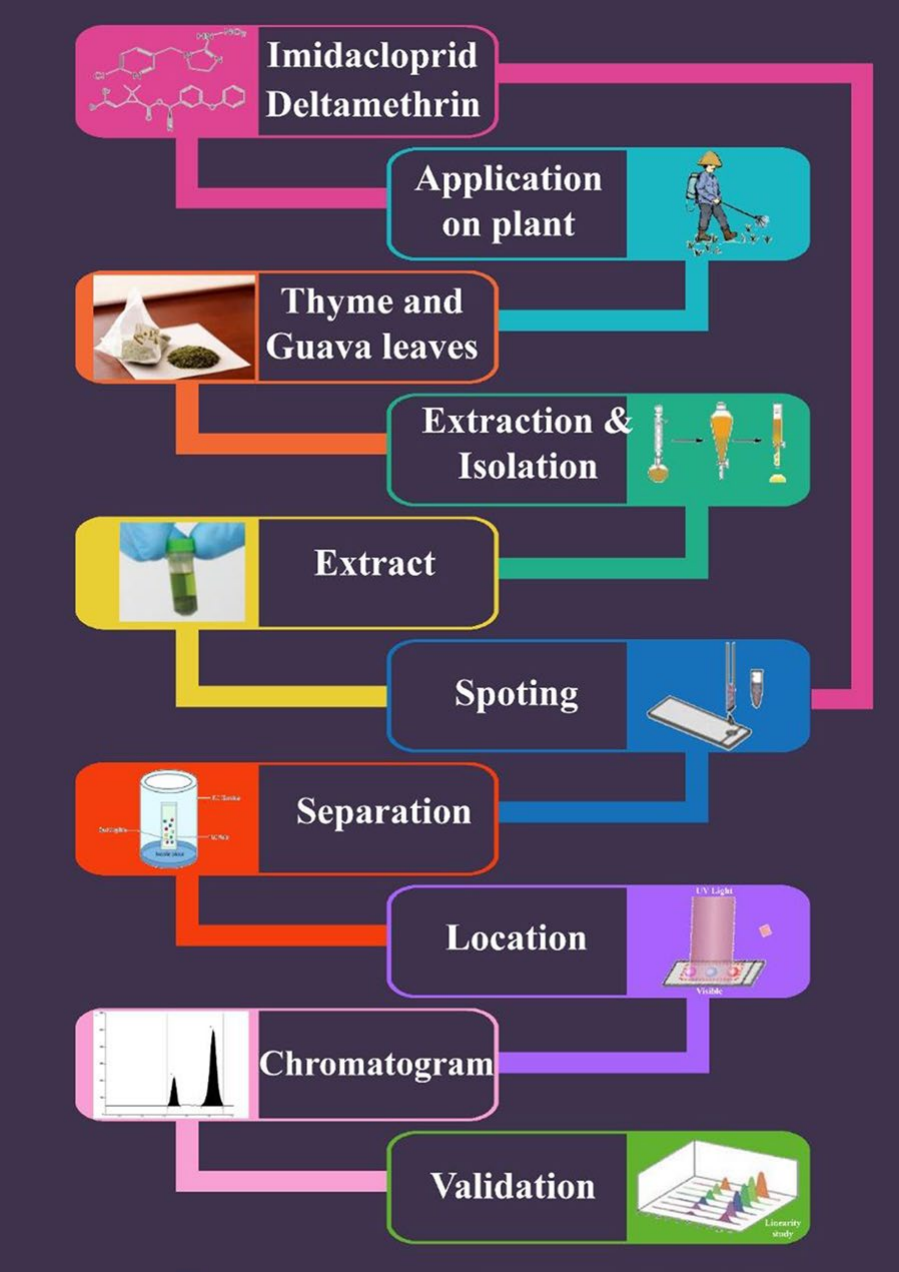

## Introduction

**Fig. 1:**
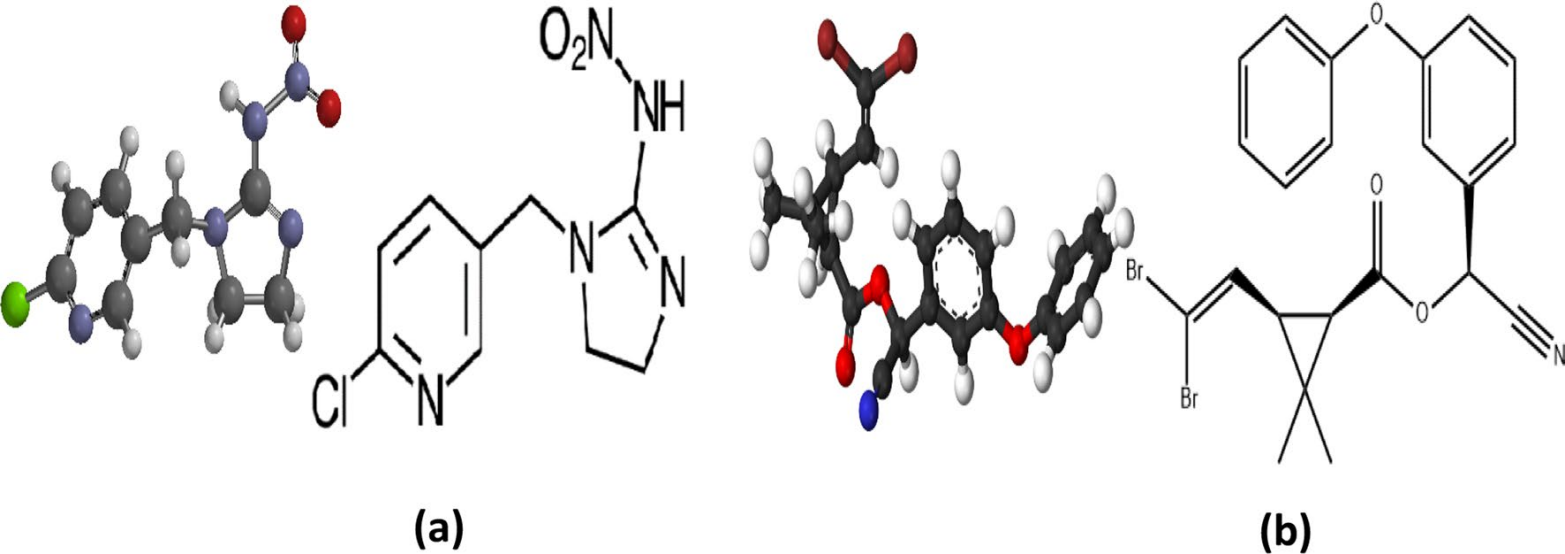
Chemical structures of (**a**) imidacloprid (**b**) deltamethrin

Nowadays, agriculture has witnessed widespread using of many pesticides to get rid of pests in order to increase production of agricultural crops. On the other hand, excessive pesticides use causes contamination of crops and this leads to pollution of the environment and human health hazards [1]. Neonicotinoids as IMD and pyrethroids as DLM are of the most currently used pesticides in the world due to their selective toxicity on the target invertebrates [2]. IMD “1-[(6-Chloro-3-pyridinyl)methyl]*N*-nitro-2-imidazolidinimine” is a systemic and contact pesticide; it acts as an agonist of acetylcholine, which can repress the acetylcholinesterase transmission by binding to postsynaptic nicotinic receptors in the insect’s central nervous system. This causes accumulation of acetylcholine, leading to the paralysis and death of insects [3] (Fig. 1a). The pyrethroid pesticide DLM, 3-(2, 2-dibromoethenyl)-2,2-dimethyl-, (S)-cyano(3-phenoxyphenyl)methyl ester, (1R,3R) Cyclopropanecarboxylic acid (Fig. 1b), is usually used for lepidopterous pests control on different crops [4]. It executes insects by contact or ingestion through distracting their nervous system. It has been commonly used to fight pests of different plants [5].

Thyme (*Thymus* *vulgaris* L.) is a widely used aromatic herb in pharmaceuticals [6] due to its antioxidants activity thus enhances immunity. Other *Thymus* spp. have used traditionally due to its pharmacological effects as expectorant, antiseptic, anthelminthic, antispasmodic, calming effects, antioxidative, antihypertensive, antirheumatic, antivirotic and antimicrobial [7].

Guava (*Psidium* *guajava*) is a phytotherapeutic plant used in folk medicine to treat many disorders like gastroenteritis, diarrhea, vomiting, coughs, toothache and sore throat [8].

In 2009, IMD was banned in the European Union (EU) due to its toxicity to honeybees even though it was considered the best seller between pesticides, and was Federal Drug Agency (FDA) approved to be used as parasite treatments for pets in the US [9].

Broad-spectrum synthetic pyrethroids as DLM were only accepted among all pesticides to protect plant damage in Finland [10], especially after 2004 when permethrin was withdrawn from the EU market due to its toxicity.

In literature, many analytical techniques have been presented for the determination of IMD such as chromatography [11, 12], fluorimetry [13], colorimetry [14], Fourier transform infrared spectroscopy (FT-IR) [15] and electrochemistry [2].

While DLM residues were quantitated by various methods in different matrices such as chromatography [16, 17] enzyme-linked immunosorbent assay (ELISA) [18] and electrochemical methods [19].

IMD and DLM were extracted and purified from plant materials using different protocols such as solid phase extraction [20, 21] and supercritical fluid extraction [22, 23]. In this study, extraction and clean-up of IMD and DLM residues in samples was done using QuEChERS method, an original non-buffered method which involves two steps: A liquid–liquid extraction and dispersive solid-phase extraction clean-up, thus provide a simple non-expensive cleanup method [24].

The current work aims to develop simple, time and cost-saving protocols for extraction of two dangerous but widely used pesticides namely IMD and DLM in Egyptian field from different plant parts and quantitate them to suggest the best pre-harvest intervals (PHIs) to avoid their health hazards.

This study presents for the first time TLC–densitometric method to be green, sensitive and selective for determination of IMD and a simple economical TLC–densitometric method for DLM determination. For both methods chitosan nanoparticles were used to enhance separation.

## Experimental

### Apparatus and software

The following apparatuses were used: The plates used were 10 × 20 cm, coated with 0.25 mm silica gel 60 F_254_ (Merck, Germany). The samples were applied to the plates using a CAMAG Linomat 5autosampler (CAMAG, Switzerland) with 10 μL micro-syringe. CAMAG TLC scanner model 3S/N 1302139 with winCATS software (CAMAG, Switzerland) was used for scanning. For extraction and clean-up (QuEChERS protocol), Sartorius balance; accuracy ≤ 0.001 g (Göttingen, Germany), Snijders vortex (Tilburg, Holland) and Thermo scientific Cooling centrifuge (SL 16R) (Waltham Massachusetts, USA) were used.

### Materials and reagents

#### Pure samples

- IMD standard was purchased from First Kem for Agriculture Pesticides Company (Assiut, Egypt). Its purity was certified to be 98%.
- DLM standard was purchased from Dr. Ehrenstorfer GmbH (Augsburg, Germany). Its purity was certified to be 99.5%.
- Dibutyl phthalate, used as internal standard (IS) was supplied from S.C Johnson Wax Egypt Company, (Alsharqiya, Egypt). Its purity was certified to be 99%.

#### Commercial samples

- Matador 35% SC bait (labelled to contain 35% W/V of IMD), was purchased from First Kem for Agriculture Pesticides Company (Assiut, Egypt).
- Deltathrin 25 EC (labelled to contain 2.5% W/V of DLM) was purchased from Egyptian Company for Pesticides and Chemicals (Alsharqiya, Egypt).

#### Chemicals and solvents

Used chemicals and solvents didn’t need prior purification.

Acetonitrile (HPLC grade), n-hexane, isopropyl alcohol, ethyl acetate, toluene and methanol were purchased from (Sigma, Munich, Germany).

Anhydrous magnesium sulfate and sodium chloride were purchased from El-Nasr Company for chemicals (Cairo, Egypt).

Primary secondary amine (PSA) was purchased from (Agilent Technologies Company, USA).

Chitosan was purchased from (Sigma Aldrich, Belgium).

### Environmental sample (field application and sampling)

Field treatment was carried out in sections at Egyptian drug authority, Egypt. IMD (Matador 35% SC bait) and DLM (Deltathrin 25 EC) were applied to thyme and guava leaves. One section was left without treatment to be used as a control. Both treated and untreated samples of thyme and guava leaves were collected in random manner in 3 replicates at various intervals (0, 1, 2, 5, 8, 12 and 16 days) after application of pesticides. Collected leaf samples were then granulated into fine powder and kept in refrigerator at 2–4 °C till residues analysis.

### Standard solutions

Methanol was used as a solvent to prepare IMD and DLM standard stock solutions with concentration (1 mg/mL) as well as, dibutyl phthalate (IS) stock solution with concentration (5 mg/mL).

IMD and DLM working solutions were then prepared from their stock solutions by transferring serial dilutions of both pesticides in methanol to obtain concentration range of (20–220) µg/mL for IMD and (20–240) µg/mL for DLM. IS (dibutyl phthalate) was then added for each dilution to reach to final concentration 1000 µg/mL of internal standard.

### Synthesis of chitosan nanoparticles

**Fig. 2:**
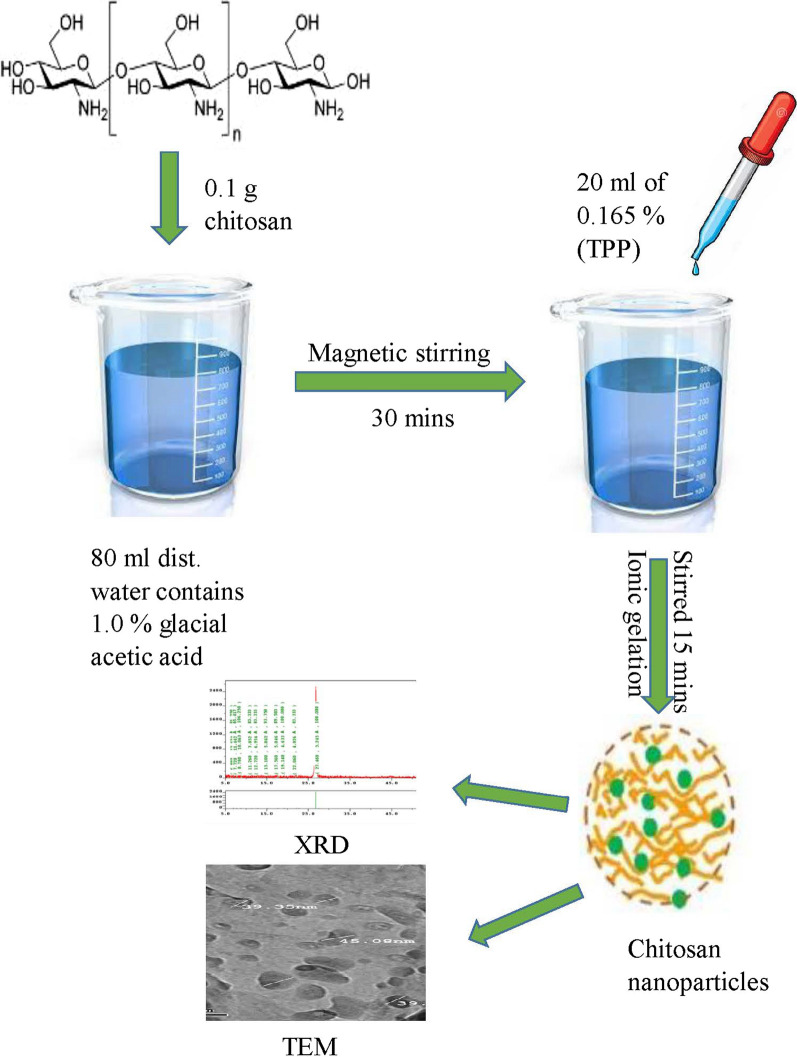
synthesis of chitosan nanoparticles

Chitosan (abbreviated as ChT) solution was prepared by dissolving 0.1 g of ChT in 80 mL distilled water contains 1.0% glacial acetic acid then dissolve for 30 min using magnetic stirring. Once dissolved, 20 mL of 0.165% Sodium tripolyphosphate (TPP) solution was added drop wise to the ChT solution and the mixture was stirred for additional 15 min. The formation of ChTNPs started spontaneously via the initiation of ionic gelatin mechanism induced by TPP (Fig. 2). The formed ChTNPs were stored at 2–8 °C [25].

### Preparation of nanochitosan TLC plate

First impregnation of TLC plate was carried out by dipping plate into ChTNPs 0.5% solution, allowing its developing in an ascending manner till saturation of the plate, then the plate was left air dried overnight.

### Chromatographic conditions

Then chromatographic separation was performed on TLC aluminum sheet (10 cm × 20 cm) coated with silica gel 60 F_254_ previously impregnated by ChTNPs 0.5% as the stationary phase. For IMD, the developing was imparted using developing system consisted of isopropyl alcohol while, for DLM, *n*-hexane‒toluene‒ethylacetate (7:3:1, v/v/v) was used as the developing one. For both methods, the solutions of two pesticides have been put as separate spots at distance 1.5 cm away from the end of plates.

Chromatography chamber saturation was performed separately for 30 min for each using isopropyl alcohol for IMD and n-hexane‒toluene‒ethylacetate (7:3:1, v/v/v) for DLM before development. Developing of normal phase TLC plates were over 8 cm, then air dried and specifically scanned at 270.0 nm for IMD and 230.0 nm for DLM.

## Procedure

### Calibration curve construction

Using a micro-syringe, fixed volumes (10 μL) of various concentrations of working solutions were spotted on three TLC plates, and then analyzed under mentioned chromatographic conditions covering the range of 0.2–2.2 μg/spot for IMD, and 0.2‒2.4 μg/spot for DLM. Calibration curves have been created through plotting the relevant concentrations against the peak area, then regression equations were calculated for the pesticides studied and used to determine concentrations of unknown samples.

### Method validation

Accuracy—Proposed methods accuracy was calculated by measuring three different concentrations within the specified range for three times, for IMD concentrations used were (0.5, 0.7 and 1.0 µg/spot), while for DLM were (0.25, 0.35 and 0.45 µg/spot).

Precision—Repeatability (intraday) was calculated by measuring the response of three concentrations within the specified range for each standard repeated three times within the day while intermediate precision (interday) was assessed by measuring the response of three concentrations within the specified range repeated three times in three successive days then RSD was then computed, for IMD concentrations used were (0.2, 1.0 and 1.4 µg/spot), while for DLM were (0.6, 1.0 and 1.4 µg/spot).

Limits of detection and quantification—by applying the formula: LOD = 3.3 (s/S), LOQ = 10 (s/S), as “s” and “S” are standard deviation of intercept and slope of the calibration curve, respectively.

Robustness—was carried out by making small changes in the chromatographic conditions, volume of mobile phase and duration of saturation of chromatography chamber.

### Analysis of commercial samples solutions

- A volume of Matador SC bait containing 100.0 mg of IMD was completed to 100 mL with methanol to obtain 1.0 mg/mL stock solution.
- For DLM, a volume of Deltathrin 25 EC, claimed to contain 25.0 mg was completed to 25 mL with methanol to obtain 1.0 mg/mL stock solution.

These stock solutions were used to prepare working solutions of each pesticide having concentration within the linear range. Dibutyl phthalate has been added to each working solution to obtain final concentration of 1000 µg/mL of internal standard. The volume was completed with methanol for each solution.

The procedures mentioned under construction of calibration curve were applied to the commercial samples. By applying corresponding regression equations, concentrations of IMD and DLM in their commercial samples were calculated.

### Extraction and analysis of environmental samples

IMD and DLM residues extraction and clean-up was carried out by applying QuEChERS protocol [24]. 1.0 g of frozen homogeneously crushed samples were weighed and mixed with 4.0 mL each of distilled water then 3.0 mL of acetonitrile and 0.1 mL of dibutyl phthalate were added to centrifuge tube. The tube was then recapped and vortexed for 30 s. 0.4 g of anhydrous MgSO_4_ and 0.1 g of NaCl were added to centrifuge tube that was closed and shaken well, then centrifuged at 4000 rpm for 5 min for separation of solid materials from the liquid layers.

Purification and removal of excess residual water was performed using a rapid dispersive solid phase extraction (d-SPE) method (a sorbent-based technique widely used in sample preparation for purification of both samples) in which 150.0 mg of MgSO4 and 50.0 mg of PSA were added to the transferred aliquot of top acetonitrile layer then was vortexed for 1 min.

The tube was then centrifuged at 4000 rpm for 2 min for separation of solid materials from the liquid layer.

## Results and discussion

In this work, we concerned with developing a simple and economic method for selective detection of IMD and DLM residue levels in real samples without any interference. TLC separation is known to be enhanced by impregnation [26]. Nanoparticles have proven excellent results in this context [27]. One of the most widely spread, naturally occurring, available, and non-expensive nanoparticles are those derived from chitin. Chitosan (ChT), is a cationic biopolysaccharide derived from chitin [28].

### Morphology of chitosan nanoparticles

**Fig. 3:**
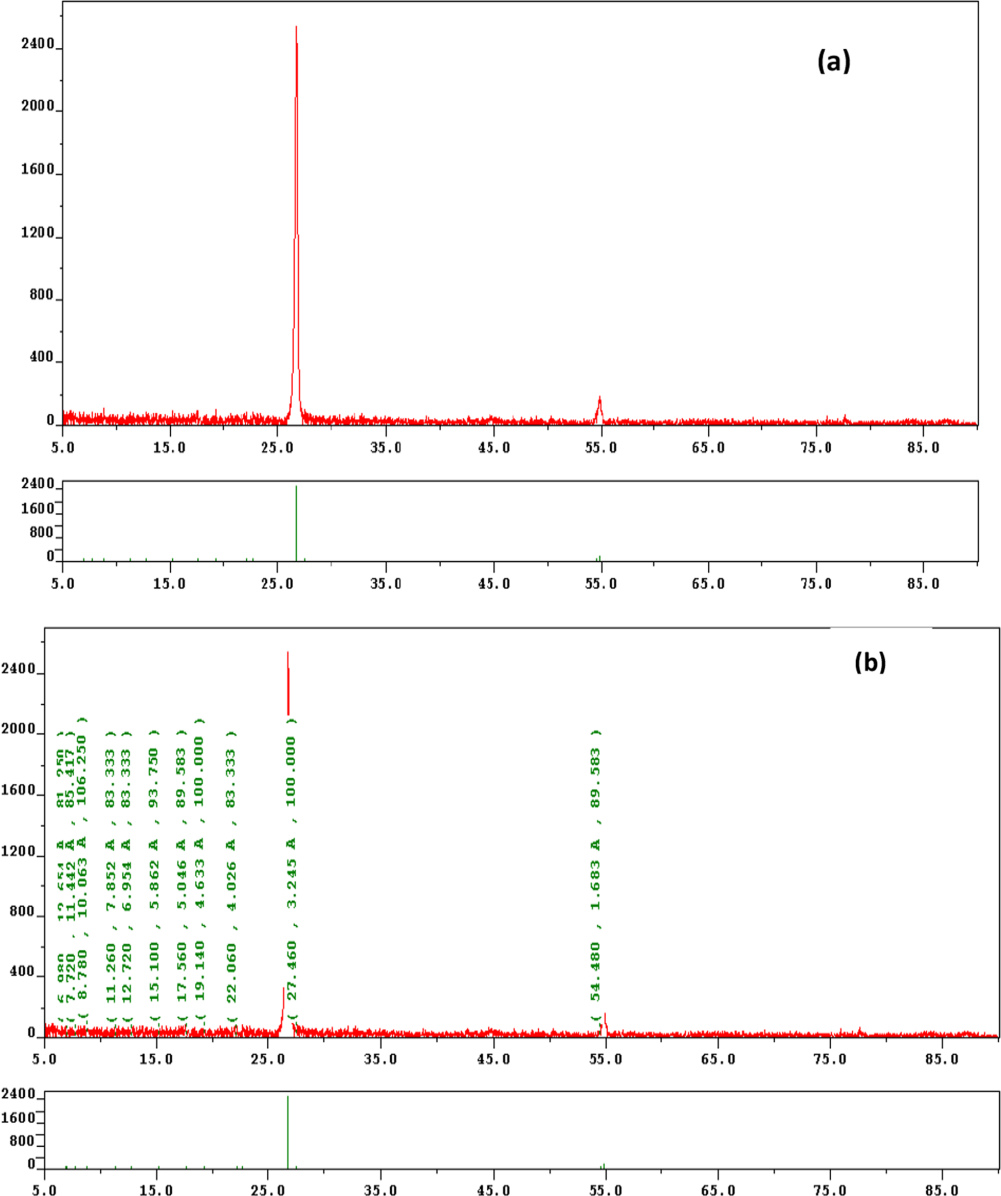

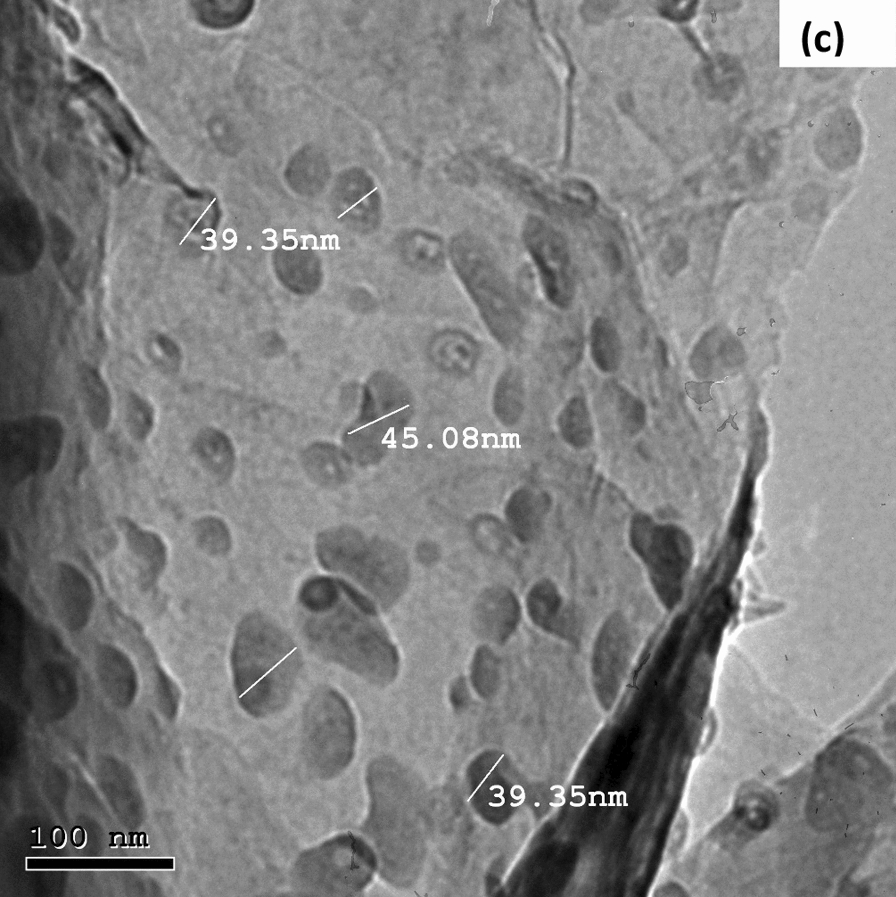
**a**, **b** XRD pattern of ChTNP. **c** Micrographs of ChTNP using TEM

Synthesized ChTNP patterns were recorded by X-ray powder diffraction and shown in Figs. 3a, b. Chitosan showed a characteristic crystalline peak at 2θ = 27.4°, which was slightly shifted to a higher diffraction angle, which indicates better crystalline nature of chitosan. XRD analysis showed crystallization of chitosan from shells of shrimp and there was noticeable peaks appeared for chitosan.

TEM was used to study morphology of the ChTNP. Figure 3c showed a characteristic morphology of synthesized ChT nanoparticles.

TEM image showed a uniform spherical shape of nanoparticle smooth surface. ChT nanoparticle diameter was measured found to be around 39–46 nm.

### Method development

#### Developing system

Chitosan (ChT), a cationic biopolysaccharide derived by deacetylation of chitin [28]. ChT contains many active sites such as amine group and – OH group. These reactive groups allow ChT to be easily transformed into gels, films, nanofibers, and nanoparticles. The variety of reactive groups can interact with compounds through chemical or physical adsorption, anion–cation interactions and electrostatic interactions [29].

ChTNPs have the properties of both ChT and nanoparticles such as small size, surface and interface effect and quantum size effects.

Using ChTNPs impregnated on TLC aluminum sheet coated with silica gel, the separation of IMD and DLM was enhanced. The interaction between ChTNPs and silica is supposed to be due to linking between protonated amino groups of chitosan polymer units and dissociated hydroxyl ones on silica surface [30]. Interaction with the drugs was through attraction between active hydroxyl groups of the stationary phase (in both free groups in silica and polymer units moiety) and functional groups in the pesticides’ molecules.

The most critical step in TLC method development is usually finding the optimal solvent system.

Many developing systems were tried on nanochitosan impregnated TLC plates for both drugs such as for IMD systems were methanol: ammonia 3%, methanol: ammonia: *n*-hexane, toluene: methanol: chloroform: ammonia and chloroform: cyclohexane: acetic acid.

But for DLM tried systems were *n*-hexane: ethylacetate, *n*-hexane: toluene, *n*-hexane: chloroform and petroleum ether: ethanol: glacial acetic acid but all these systems but did not improve the separation.

**Fig. 4:**
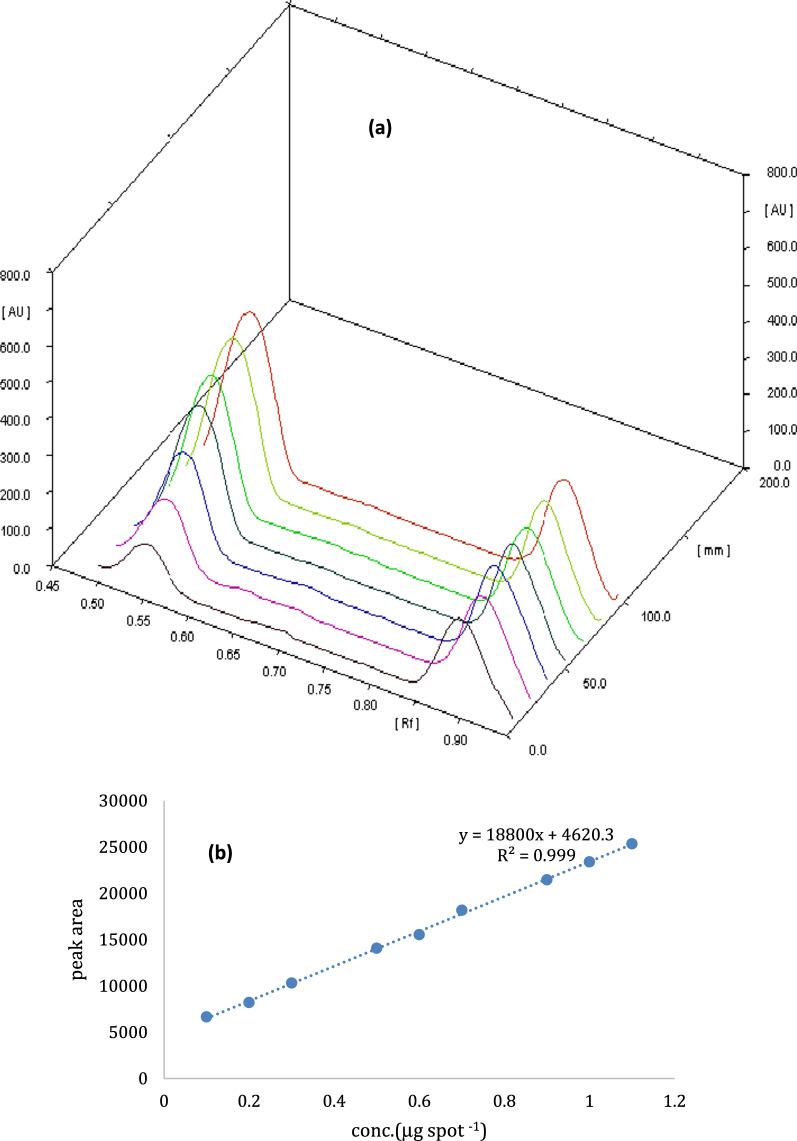

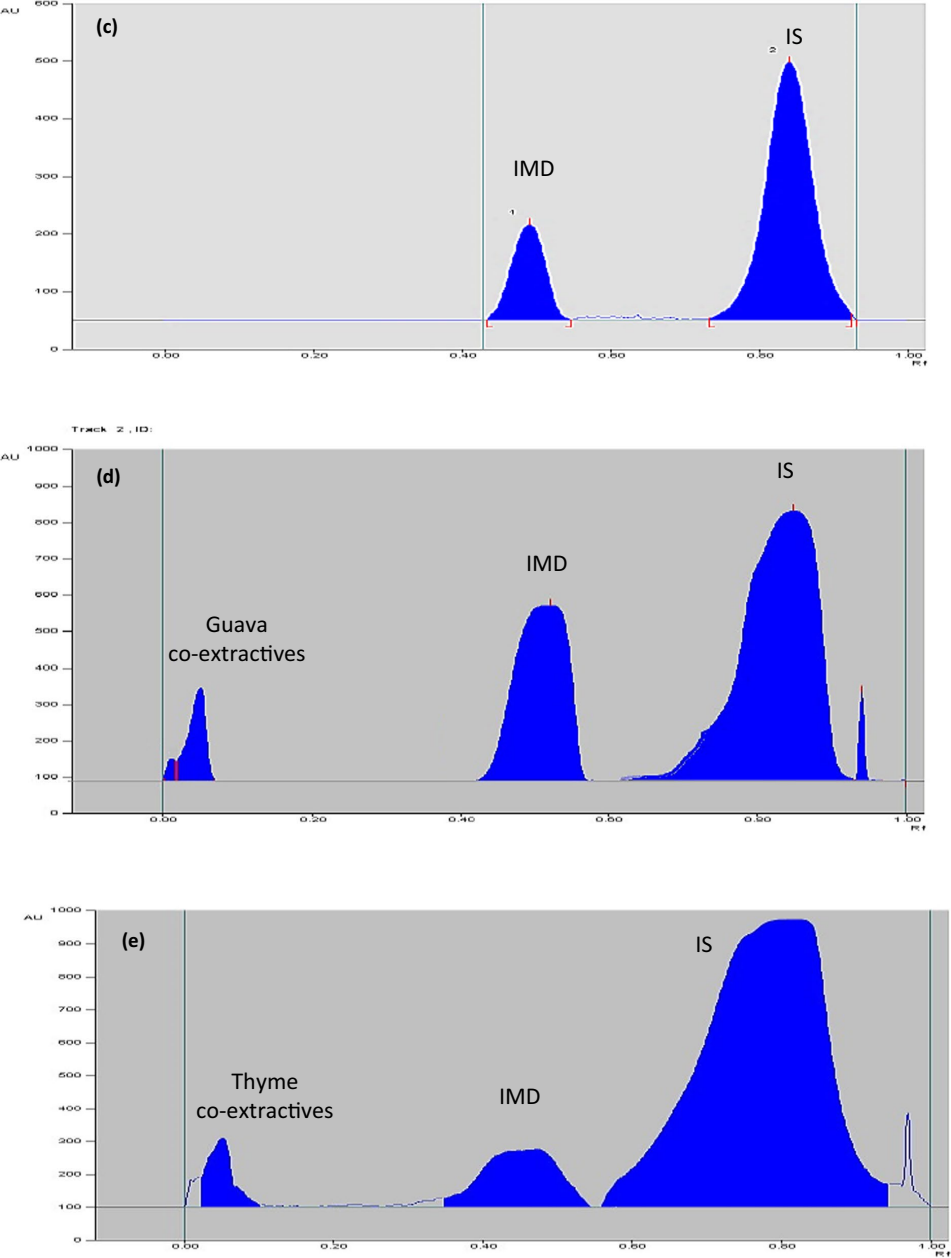
**a** Three dimensions TLC-densitometric chromatogram of serial dilutions of IMD (*R*_*F*_ 0.51) with constant concentration of IS (*R*_F_ of 0.89) at wavelength 270 nm. **b** relationship between the peak area and different concentrations of IMD. **c** Two dimensions TLC-densitometric chromatogram of IMD in commercial sample. **d** Two dimensions TLC-densitometric chromatogram of guava leaves extract with IMD residue after 16 days of applying of IMD. **e** Two dimensions TLC-densitometric chromatogram of thyme extract with IMD residue after 16 days of applying of IMD

In order to achieve our goal, we have taken into account that an effort should be made to achieve a system of environmentally friendly solvent without reducing the analytical performance. Separation of IMD was successfully reached using an eco-friendly developing system without affecting the analytical performance that consisted of isopropyl alcohol where R_F_ values of IMD was 0.51 and that of dibutyl phthalate (IS) was 0.89. This is followed by densitometric detection at 270.0 nm (Fig. 4).

**Fig. 5:**
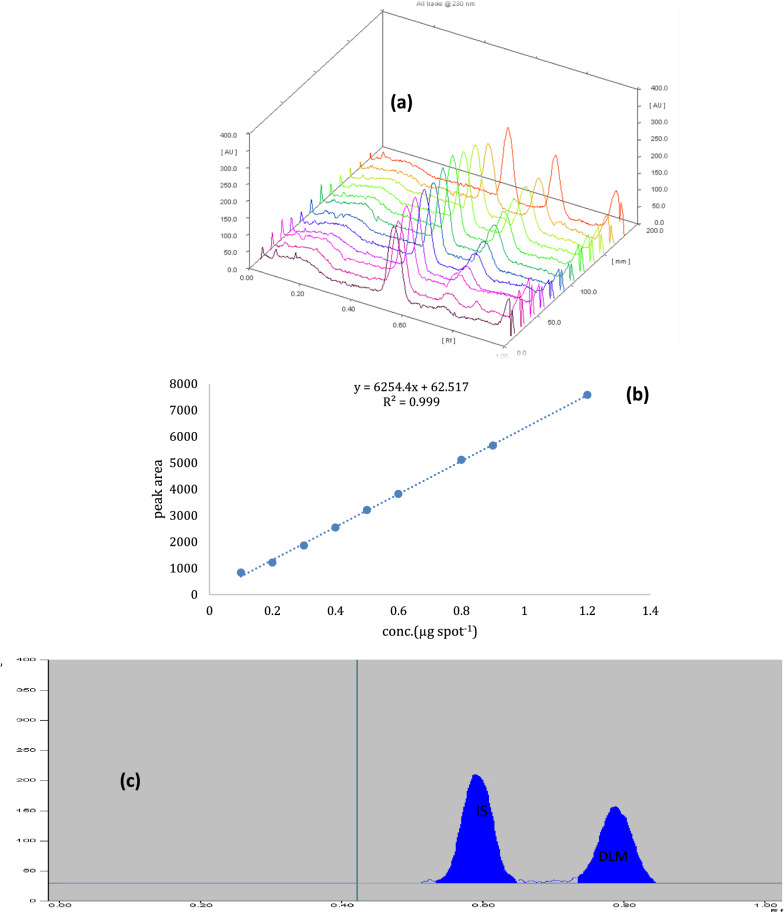

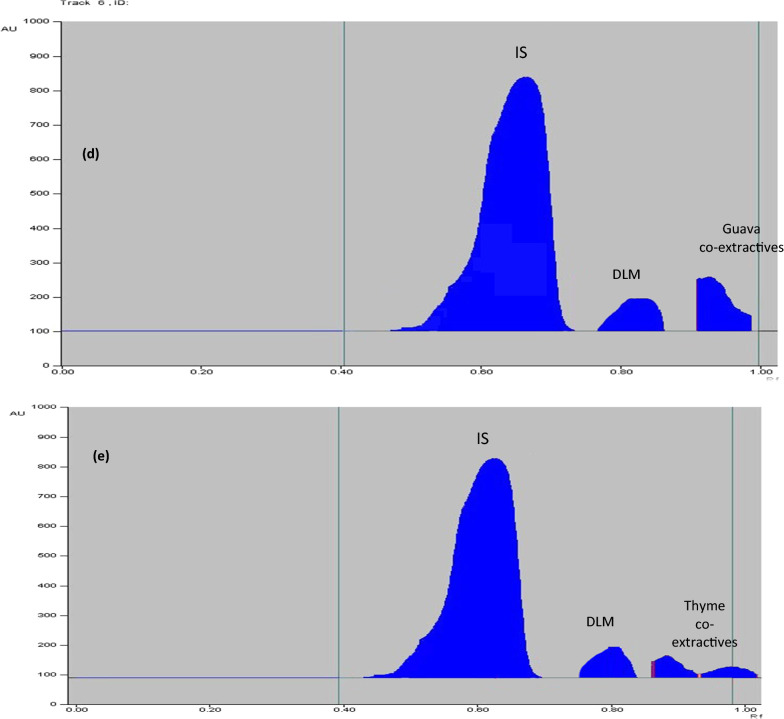
**a** Three dimensions TLC- densitometric chromatogram of serial dilutions of DLM (RF 0.80) with constant concentration IS (*R*_F_ of 0.61) at wavelength 230 nm. **b** Relationship between the peak area and different concentrations of DLM. **c** TLC-densitometric chromatogram of DLM in commercial sample. **d** Two dimensions TLC-densitometric chromatogram of guava leaves extract with DLM residue after 16 days of applying of DLM. **e** Two dimensions TLC-densitometric chromatogram of thyme extract with DLM residue after 16 days of applying of DLM

Separation of DLM from other interfering substances was done using developing system consisting of n-hexane: toluene: ethylacetate (7:3:1, V/V) with R_F_ values 0.61 for dibutyl phthalate (IS) and 0.80 for DLM at wavelength 230.0 nm (Fig. 5).

#### Scanning wavelength

Different scanning wavelengths have been tested; the optimal wavelength with symmetrical peaks, higher sensitivity, and lowest noise was at 270.0 nm for IMD and 230.0 nm for DLM. The scanning light beam slit dimensions was selected to ensure full coverage of spot dimensions on the scanned track. Various slit dimensions have been tried, highest sensitivity obtained at slit dimensions 3 mm × 0.45 mm, with scanning rate 20 mm/s.

#### Validation parameters

The proposed methods have been validated according to the guidelines of the International Conference on Harmonization (ICH) [31]. The linearity of the methods has been verified, and the calibration curves have been created. By plotting the pesticides peak area against the concentrations of IMD and DLM, a good correlation was obtained. In concentration range of 0.2–2.2 µg/spot for IMD and 0.2–2.4 µg/spot for DLM, linear response was obtained (Figs. 4, 5).

The regression equations have been found to be:

$$ {\text{For\,IMD}}:Y = { 188}00X + {462}0.{3};r = \, 0.{999}; $$

$$ {\text{For\,DLM}}:Y = {6254}.{4}X + {62}.{517};r = 0.{999}, $$

knowing that *Y,* *X* *and* *r* represent the peak area, the concentration in µg/spot and the correlation coefficient, respectively.

Summary of the validation parameters are presented in Table 1. Robustness of the methods have verified by deliberate small changes in the chromatographic conditions, % RSD of each pesticide has calculated; results are listed in Table 2.

**Table 1:** Validation parameters for developed TLC -densitometric methods

Parameter	IMD	DLM
Range(μg/spot)	0.2–2.2	0.2–2.4
Accuracy^a^ [mean% ± SD]	100.49 ± 1.62	100.57 ± 0.39
Precision^b^ (%RSD)		
Repeatability(intraday)	1.92	1.39
Intermediate(interday)	1.92	1.92
LOD^c^ [μg/spot]	0.002	0.00116
LOQ^c^ [μg/spot]	0.0054	0.0035
Linearity		
Slope	18,800	6254.4
Intercept	4620.3	62.517
Correlation coefficient(r)	0.999	0.999
SE	227.881	76.976

^a^Mean of minimum nine determinations over a minimum of three concentration levels covering the specified range

^b^Repeatability (intraday) was assessed by measuring the response of three concentrations within the specified range for each standard repeated three times within the day and intermediate precision (interday) was assessed by measuring the response of three concentrations within the specified range for each standard repeated three times in three successive days, for IMD concentrations used were (0.2, 1.0 and 1.4 µg/spot), while for DLM were (0.6, 1.0 and 1.4 µg/spot)

^c^LOD and LOQ can be calculated according to the formula: LOD = 3.3 (*s*/*S*) and LOQ = 10 (*s*/*S*)

**Table 2:** Robustness assessment of the developed TLC–densitometric methods for determination of IMD and DLM

Robustness parameter	SD of peak area		% RSD	
	IMD	DLM	IMD	DLM
(1) Volume of mobile phase				
98 ml	144.07	100	2.87	2.78
100 ml	85.26	31.18	1.68	0.83
102 ml	122.46	40.40	2.48	0.97
(2) Duration of saturation				
25 min	296.45	26.93	1.46	1.43
30 min	227.33	11.89	1.11	0.61
35 min	297.58	37.55	1.60	1.97

System suitability parameters including retention factor, resolution of peaks, selectivity factor and tailing factor were computed for the proposed methods and results were satisfactory as presented in Table 3.

**Table 3:** Parameters of system suitability for proposed TLC-denstiometric methods for the determination of IMD and DLM

Parameter	IMD	DLM	Reference value (USP)
Retention factor (R_F_)	0.51	0.80	–
Resolution (Rs)^a^	4	2.69	R > 1.5
Selectivity (α)^a^	1.68	1.37	α > 1
Tailing factor (T)	1.1	1	T ˂ 2

^a^Between drug peak and that of internal standard

### Analysis of commercial samples

The proposed methods were efficient and applicable to determine IMD and DLM in their commercial products Matador 35% SC bait and Deltathrin 25 EC without any interference from other matrices as displayed in Figs. 4b and 5b.

To ensure validity of the proposed methods, standard addition technique was applied and good recovery was obtained as shown in Table 4.

**Table 4:** Recovery of the developed TLC-denstiometric methods in pure form and application of standard addition technique

IMD			DLM		
Taken µg/spot	Found µg/spot	Recovery %	Taken µg/spot	Found µg/spot	Recovery %
0.3	0.30058	100.19	0.5	0.505	101.01
0.7	0.71572	102.25	0.7	0.7035	100.50
0.9	0.8914	99.04	0.9	0.90199	100.22
1.4	1.4543	103.87	1.2	1.2011	100.09
1.8	1.7914	99.52	1.6	1.5836	98.96
Mean		100.97	Mean		100.1518
SD		2.037	SD		0.755
RSD%		2.01	RSD%		0.0075

Product	Standard addition			
	Claimed taken µg/spot	Added µg/spot	Found µg/spot	Recovery %
Standard addition technique				
IMD in Matador 35% SC bait	0.80	0.40	0.41	102.50
		0.80	0.794	99.25
		1.2	1.232	102.70
	Mean			101.47
	SD			1.92
	RSD%			1.89
DLM in Deltathrin 25 EC	2.0	0.40	0.414	103.45
		1.2	1.212	101.00
		2.0	2.08	104.00
	Mean			102.80
	SD			1.597
	RSD%			1.553

### Analysis of IMD in guava leaves and thyme extract

Separation of IMD residues from guava leaves and thyme extracts was accomplished by proposed method and good chromatographic separation was obtained as displayed in Fig. 4c, d.

**Fig. 6:**
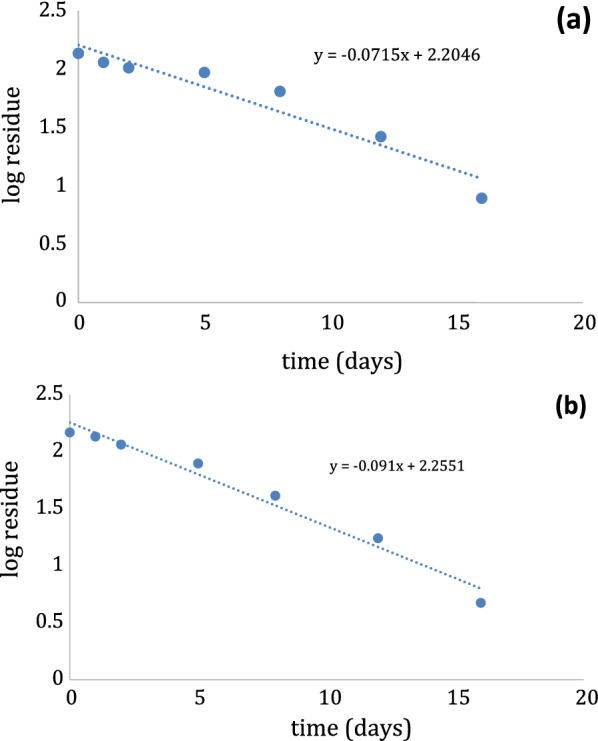
Logarithmic values of decline of residues of (**a**) IMD in guava leaves and (**b**) IMD in thyme leaves with time using the proposed method

Concentrations of IMD residues in beforehand treated leaves of thyme and guava over the period of 0, 1, 2, 5, 8, 12 and 16 days presented in Table 5. Preliminary concentrations of IMD residues were 147.31 mg/kg and 135.90 mg/kg in thyme and guava leaves, respectively. 16 days later from pesticide application, residues concentrations of IMD decreased to 4.71 mg/kg and 7.80 mg/kg in the leaves of thyme and guava, respectively. After 16 days, only 3.20% and 5.74% of the preliminary concentrations were detected in thyme and guava leaves. IMD half-life values (t_1/2_) have been calculated and found to be 7.62 days for thyme and 9.70 days for guava leaves (Fig. 6).

**Table 5:** IMD and DLM residues levels in thyme and guava leaves

Pesticide name	Thyme leaves					Guava leaves			
	Time after application	Residue concentration mg/kg ± S.D	Log Residue	Persistence%	Loss%	Residue concentration mg/kg ± S.D	Log Residue	Persistence%	Loss%
IMD	Zero time 1 day 2 days 5 days 8 days 12 days 16 days	147.31 ± 0.21 135.49 ± 0.52 115.16 ± 0.44 78.52 ± 0.16 41.03 ± 0.05 17.39 ± 0.03 4.71 ± 0.02	2.17 2.13 2.06 1.89 1.61 1.24 0.67	100 92.1 78.17 53.36 27.85 11.80 3.20	00.00 8.02 21.82 46.64 72.15 88.19 96.80	135.90 ± 0.10 113.61 ± 0.029 102.31 ± 0.018 93.23 ± 0.028 64.06 ± 0.165 26.27 ± 0.133 7.80 ± 0.039	2.13 2.06 2.01 1.97 1.81 1.42 0.89	100 83.6 75.28 68.6 47.14 19.33 5.74	00.00 16.40 24.72 31.40 52.86 80.67 94.26
	t_1/2_^a^	7.62 days				9.7 days			
DLM	Zero time 1 day 2 days 5 days 8 days 12 days 16 days	178.39 ± 0.27 156.51 ± 0.37 133.50 ± 0.11 98.97 ± 0.17 93.97 ± 0.13 45.88 ± 0.079 10.39 ± 0.14	2.25 2.19 2.12 1.99 1.97 1.66 1.02	100 87.73 74.84 55.48 52.68 25.72 5.82	00.00 12.26 25.16 44.52 47.32 74.28 94.18	180.75 ± 0.18 131.58 ± 0.13 112.45 ± 0.11 112.10 ± 0.16 98.15 ± 0.09 72.67 ± 0.13 11.55 ± 0.09	2.26 2.12 2.05 2.049 1.99 1.86 1.06	100 72.79 62.21 62.02 54.30 40.20 6.39	00.00 27.20 37.79 37.98 45.70 59.80 93.61
	t_1/2_^a^	10.28 days				12.1 days			

^a^The half-life (t_1/2_) is calculated from k (elimination rate constant) by formula: *t*_1/2_ = ln 2/*K*

In coherence with EU, 2016 guidelines (European Commission, European Union Pesticides Database, 2016) MRL value of IMD on thyme and guava leaves is 2 mg/kg and 0.05 mg/kg, respectively. Noteworthy, applying the proposed method showed that the IMD level was still above the MRL after 16 days, concluding that the PHI was estimated to be above 16 days, about 3 weeks to reach the accepted concentration limit of IMD to avoid harmful health effect.

### Analysis of DLM in guava leaves and thyme extract

Determination of DLM residues concentration in pretreated samples of thyme and guava leaves was also accomplished by applying proposed method and good chromatographic separation was obtained as displayed in Fig. 5c, d.

**Fig. 7:**
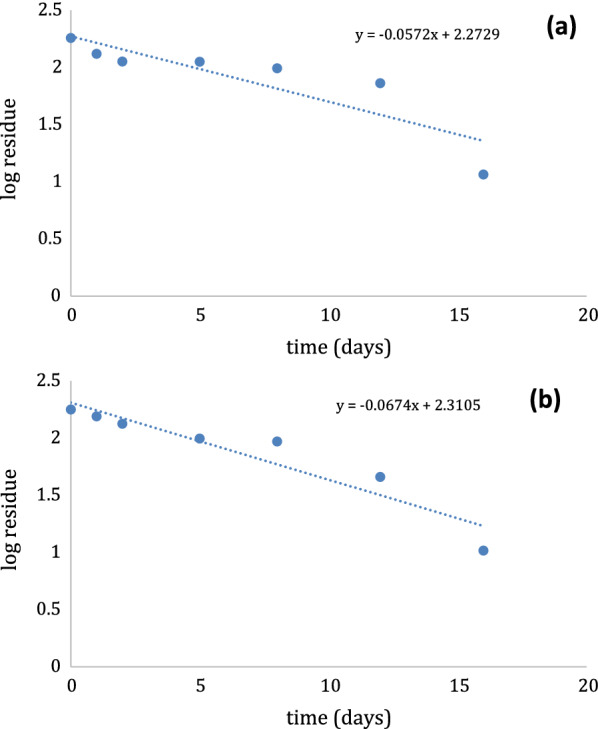
Logarithmic values of decline of residues of (**a**) DLM in guava leaves and (**b**) DLM in thyme leaves with time using the proposed method

Concentrations of DLM residues in pretreated leaves of thyme and guava over the period of 0, 1, 2, 5, 8, 12 and 16 days presented in Table 5. Preliminary concentrations of DLM were 178.39 mg/kg in thyme and 180.75 mg/kg in guava leaves. Concentrations of DLM residues decreased to 10.39 mg/kg and 11.55 mg/kg in thyme and guava leaves respectively, 16 days later from pesticide application. After 16 days, only 5.82% and 6.39% of the preliminary concentration were detected in thyme and guava leaves. DLM half-life values (t_1/2_) have been calculated and found to be 10.28 days for thyme and 12.1 days for guava leaves (Fig. 7).

As stated by EU, 2016 guidelines [32], MRL value of DLM in thyme and guava leaves 2 mg/kg and 0.01 mg/kg, respectively. Noteworthy, applying the proposed method showed that the DLM level was still above the MRL after 16 days (Table 5), concluding that the PHI was estimated to be above 16 days, about 3 weeks to reach the accepted concentration limit of DLM to avoid harmful health effect.

### Analytical eco‑scale greenness evaluation of proposed methods

Eco-scale investigation is a semi-quantitative method used for testing greenness of analytical procedures [33]. Eco-scale tool depends on penalty point from a base of 100 (the perfect score of the green analysis method). All penalty points are collected and then subtracted from 100 per parameter (nature and amount of reagents, energy consumed, occupational hazard and waste produced) [34]. Higher score indicates greener, more environmentally friendly and cost-effective analytical procedure. A green analysis is considered excellent if eco-scale score more than 75, acceptable if more than 50, and if less than 50 supposed inadequate [35]. Eco-Scale score was computed for the suggested methods and the reported methods, and the results proved that the proposed TLC method for IMD exceled over the reported TLC method [36] as a greener alternative for determination of IMD, as in the reported TLC-densitometric method mobile phase used was consisting of toluene: acetonitrile (7:3 v/v) and detection at 270.0 nm. For DLM, although the eco-scale score of the proposed method was less than eco- scale score of the reported method [37], the proposed method is still considered ecofriendly as the green analysis is considered excellent if eco-scale score more than 75. The results were summarized in Table 6. Moreover, the previous paper didn’t specify which vegetable on which they apply their method or test the suitable pre-harvest time which is the main target of our research as our work concerns with studying kinetics of decline rate of both pesticides in thyme and guava leaves, estimating t_1/2_ and the proper PHI. Noteworthy, the novelty of our work was mainly enhancing separation and sensitivity of the proposed methods by modification of TLC plates by synthesized ChTNPs as the developed methods succeeded to detect both pesticides without interference of other matrices and their residues in thyme and guava leaves extract without interference from active constituent of thyme (thymol) and active constituent of guava leaves (pinene).

**Table 6:** Penalty points (PPs) for the proposed TLC methods and reported methods

Parameters	Penalty points [PPs]			
	Proposed method for IMD	Reported method for IMD [36]	Proposed method for DLM	Reported method for DLM [37]
Reagents				
Isopropyl alcohol	2.0	‒	‒	‒
Toluene	‒	6.0	6.0	6.0
Acetonitrile	‒	4.0	‒	‒
*n*-hexane	‒	‒	8.0	8.0
Ethylacetate	‒	‒	4.0	‒
Water	‒	‒	‒	‒
Instrument				
Energy [> 0.1 kWh per sample]	1.0	1.0	1.0	1.0
Occupational hazard	0.0	0.0	0.0	0.0
Waste Total PPs Analytical eco-scale score	5.0 Ʃ8.0 92.0 Excellent green Analysis	5.0 Ʃ16.0 84.0 Green Analysis	5.0 Ʃ24.0 76.0 Green Analysis	5.0 Ʃ20.0 80.0 Greener Analysis

### Statistical comparison

Further ensuring the accuracy of the suggested method by statistically comparing data obtained data from proposed methods and reported methods using the F value and the student t-test indicating that there are no significant differences; suggesting that the proposed method is precise and accurate as shown in Table 7.

**Table 7:** Statistical analysis of the results obtained by the proposed TLC methods and the reported methods for the determination of IMD and DLM in pharmaceutical preparation

Parameters	IMD		DLM	
	Method A IMD	Reported method^a^	Method B DLM	Reported method^b^
Mean^c^ [%]	100.49	99.8	100.57	98.12
SD	1.62	1.7	0.396	0.8
Variance	2.62	2.89	0.157	0.64
N	3	3	3	3
Student’s t-test ^d^ (2.78)	0.363	‒	2.05	‒
F-valued (19.0)	1.103	–	4.08	‒

^a^TLC-Densitometric method: using mobile phase, consisting of toluene: acetonitrile (7:3 v/v) and detection at 270.0 nm [33]

^b^HPLC method: using C18 column and mobile phase consisting of acetonitrile and deionized water at a flow rate of 0.8 mL/min and

detection at 210.0 nm [34]

^c^Average of 3 experiments

^d^Figures between parentheses represent the corresponding tabulated value of *t* and *F* at *p* = 0.05

## Conclusion

The modification of TLC plates by synthesized ChTNPs was successfully made-up which enhanced pesticides separation. Also in this work the green analytical method was introduced to determine IMD in pure forms, commercial samples and even to determine its residues in real environmental samples thyme and guava leaves extracts. The eco-scale was calculated for the proposed method for IMD and the reported one, taking into consideration type and amount of reagents used, instruments used, energy consumption, and waste generated, and the proposed method proved to be more environmental-friendly, with good performance and validation parameters. Furthermore, we introduce a simple, sensitive and cost-effective TLC method for the determination of DLM pure forms, commercial samples and even for the determination of its residues in real environmental samples thyme and guava leaves extracts. By applying the proposed methods we tried to estimate the pre-harvest intervals (PHIs) to avoid health hazards.

## Data Availability

All additional data are available as a Additional file.

## Abbreviations

ChTNPs: Chitosan nanoparticles
Ch: Chitosan
DLM: Deltamethrin
ELISA: Enzyme linked immunosorbent assay
EU: European Union
FDA: Federal drug agency
FT-IR: Fourier transform infrared spectroscopy
ICH: International Conference on Harmonization
IMD: Imidacloprid
IS: Internal standard
LOD: Limit of detection
LOQ: Limit of quantification
MRL: Maximum residual limit
PHI: Pre-harvest interval
PSA: Primary secondary amine
QuEChERS: Quick easy cheap effective rugged safe
R_F_: Retention factor
RSD: Relative standard deviation
TEM: Transmission electron microscopy
TLC: Thin layer chromatography
TPP: Tripolyphosphate
XRD: X-ray powder diffraction
